# The fatty acid elongase Bond is essential for *Drosophila* sex pheromone synthesis and male fertility

**DOI:** 10.1038/ncomms9263

**Published:** 2015-09-15

**Authors:** Wan Chin Ng, Jacqueline S. R. Chin, Kah Junn Tan, Joanne Y. Yew

**Affiliations:** 1 Biological Mass Spectrometry, Temasek Life Sciences Laboratory, 1 Research Link NUS, 117604 Singapore, Singapore; 2 Department of Biological Sciences, National University of Singapore, 14 Science Drive 4, 117543 Singapore, Singapore; 3 Present address: Pacific Biosciences Research Center, University of Hawai'i at Mānoa, 1993 East-West Road, Honolulu, Hawaii 96822, USA

## Abstract

Insects use a spectacular variety of chemical signals to guide their social behaviours. How such chemical diversity arises is a long-standing problem in evolutionary biology. Here we describe the contribution of the fatty acid elongase Bond to both pheromone diversity and male fertility in *Drosophila*. Genetic manipulation and mass spectrometry analysis reveal that the loss of *bond* eliminates the male sex pheromone (3*R*,11*Z*,19*Z*)-3-acetoxy-11,19-octacosadien-1-ol (CH503). Unexpectedly, silencing *bond* expression severely suppresses male fertility and the fertility of conspecific rivals. These deficits are rescued on ectopic expression of *bond* in the male reproductive system. A comparative analysis across six *Drosophila* species shows that the gain of a novel transcription initiation site is correlated with *bond* expression in the ejaculatory bulb, a primary site of male pheromone production. Taken together, these results indicate that modification of *cis*-regulatory elements and subsequent changes in gene expression pattern is one mechanism by which pheromone diversity arises.

## Abstract (web summary)

Insect behaviours are often guided by chemical signals, but little is known about how pheromone diversity evolves. Here the authors show that loss of the gene *bond* in *Drosophila* eliminates the sex pheromone CH503, while silencing it reduces the fertility of males and their conspecific rivals.

## Graphical abstract

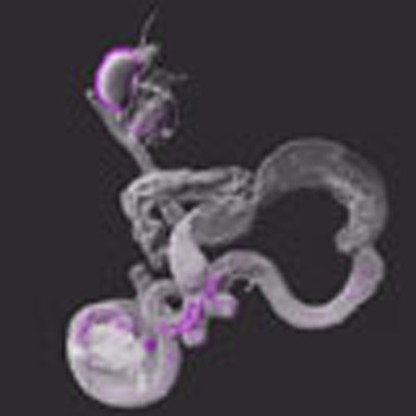

Sexual recognition in many insects is largely influenced by lipids on the cuticles, many of which function as pheromones^1^. In numerous species, changes in the pheromone profile have been implicated as a mechanism underlying reproductive isolation and speciation^2^. For example, in *Lepidoptera*^3^,^4^ and *Drosophila*^5^,^6^, allelic variations in genes encoding pheromone biosynthesis enzymes result in different sex pheromone blends and assortative mating. However, much remains unknown about the molecular mechanisms underlying the evolution of pheromone profiles.

The identification of pheromone biosynthesis enzymes is a crucial step for understanding how pheromone signals evolve. In *Drosophila*, the oenocytes and ejaculatory bulb (EB) are the major sites of pheromone synthesis^7^. In the oenocytes of *Drosophila melanogaster,* the elongation of female dienes is carried out by EloF (ref. 8). The desaturases Desat1, Desat2 and DesatF place double bonds at distinct positions in the hydrocarbon backbone of the female aphrodisiacs (7*Z*,11*Z*)-heptacosadiene and (7*Z*,11*Z*)-nonacosadiene^9^,^10^. Desat1 also contributes to the generation of characteristic male monoenes (7*Z*)-tricosene, and (7*Z*)-pentacosene^9^. Modification of the enhancer regions of DesatF is thought to underlie differences in the species and sex-specific pheromone profiles of various *Drosophila* species^11^,^12^.

In comparison, the mechanisms underlying pheromone synthesis in the EB are relatively sparse. Closely related drosophilid species exhibit large variations in the EB pheromone composition^12^,^13^. Thus, the EB serves as an excellent model for understanding the evolution of pheromone diversity. The EB is connected to the male reproductive organs and its contents are secreted exogenously to the anogenital region. In *D. melanogaster*, two male sex pheromones, 11-*cis*-vaccenyl acetate (cVA) and (3*R*,11*Z*,19*Z*)-3-acetoxy-11,19-octacosadien-1-ol (CH503), are produced in the EB^14^,^15^. Both molecules are transferred to females during courtship and function as anti-aphrodisiacs that suppress attraction from other males. To date, only one enzyme in the EB, Elo68a^16^, has been shown to contribute to the synthesis of pheromones.

In this study, we sought to identify enzymes involved in the biosynthesis of pheromones in the EB in an effort to understand the evolution of pheromone signals. We show that *jamesbond* (also known as *bond*), which encodes a very-long-chain fatty acid (VLCFA) elongase, is expressed in the EB and male reproductive organs and plays an essential role in the synthesis of the male sex pheromone CH503 and male fertility. Loss of *bond* expression results in a significant decrease in male fertility. Surprisingly, Bond also regulates the fertility of conspecific male rivals and this effect is mediated partly by CH503. A comparative analysis across six species of *Drosophila* shows that the gain of a novel transcription initiation site is correlated with a gain of *bond* expression in the EB, thus enabling the production of more complex pheromone mixtures.

## Results

### Bond is essential for the synthesis of a sex pheromone

**Figure 1:**
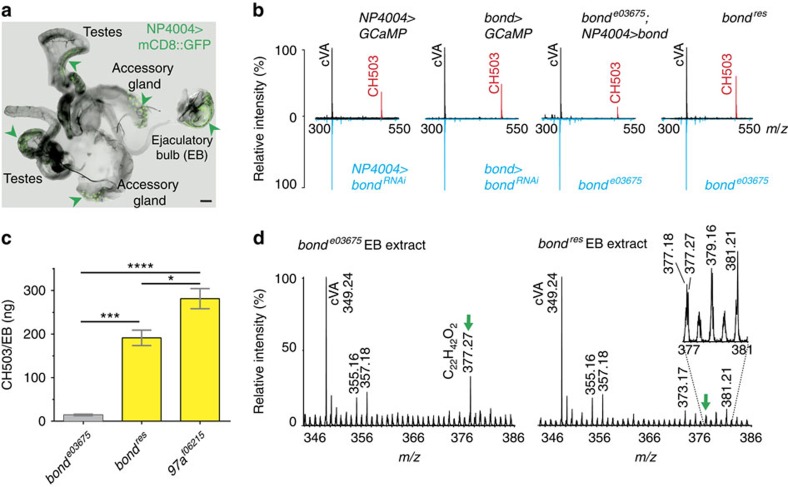
Disruption of *bond* in the ejaculatory bulb results in loss of the sex pheromone CH503. (**a**) Confocal image of *NP4004>mCD8::GFP* shows broad expression in the male reproductive organs (arrowheads); scale bar, 100 μM. (**b**) UV-LDI mass spectra recorded from the anogenital region of male flies. Signals corresponding to the mass-to-charge ratio (*m*/*z*) of cVA (*m*/*z* 349.24) and CH503 (*m*/*z* 503.38) are observed in NP400*4*>GCaMP (control) and *bond*^*res*^ males. The CH503 signal is not detected in *NP4004>bond*^*RNAi*^, *bond>bond*^*RNAi*^ *and bond*^*e03675*^ males. Overexpression of *bond* rescued CH503 production in the mutant background. Each mass spectrum was recorded from a single fly. (**c**) Quantification of CH503 by HPTLC of EB extracts reveals low levels of CH503 in *bond*^*e03675*^ males compared with *bond*^*res*^ and control line *97a*^*f06215*^. Bars represent the mean of three replicates±s.e.m. Each replicate consists of extract from 20 EBs. ANOVA with a Tukey's multiple comparison test, *P<0.05, ***P<0.001, ****P<0.0001. (**d**) UV-LDI MS spectra of EB extract from *bond*^*e03675*^ and *bond*^*res*^ flies. The signal at *m*/*z* 377.27 ([M+K]^+^; green arrow) is detected with 13-fold higher intensity in extract from *bond*^*e03675*^ compared with *bond*^*res*^. ANOVA, analysis of variance.

The elongase Bond was identified as part of a RNA interference (RNAi) screen to discover genes that contribute to the biosynthesis of pheromones in the EB. The *Drosophila* GAL4-UAS transgene expression system^17^ was used to perform RNAi-mediated knockdown in a tissue-specific manner. Using the *NP4004-GAL4* driver, which expresses in the male reproductive organs, EB, gut and parts of the central nervous system (Fig. 1a; Supplementary Fig. 1), the expression of candidate genes was silenced specifically in these organs and the lipid profiles assessed with ultraviolet laser desorption ionization mass spectrometry (UV-LDI MS). UV-LDI MS is a recently introduced method for cuticular lipid analysis that provides spatially resolved chemical profiles of intact insects^18^,^19^.

In wild-type males, the mass spectral profiles of the anogenital region (Fig. 1b) and dissected EB (Supplementary Fig. 2) consist primarily of two signals corresponding to cVA and CH503. In contrast, direct mass spectrometry analysis of dissected testes and accessory glands showed no detectable signal for cVA or CH503 (Supplementary Fig. 2), indicating that the pheromones are produced primarily in the EB. Following RNAi-mediated knockdown of *bond*, the signal for CH503 was no longer detected (Fig. 1b). Since the *NP4004* driver could represent the expression pattern of several genes proximal to *bond*, we generated a GAL4 line targeting the *bond* enhancer region. *bond-GAL4* expression is evident in parts of the gut, testes and EB (Supplementary Fig. 1). Silencing *bond* expression with the *bond* driver also eliminated the signal for CH503 (Fig. 1b). The loss of CH503 in the EB was also observed when *bond* transcription is interrupted in a PBac insertion line, *bond*^*e03675*^. Notably, the defect was rescued upon excision of the PBac insertion (*bond*^*res*^) or overexpression of *bond* in the *bond*^*e03675*^ background (Fig. 1b).

To test whether the loss of CH503 signal could be due to a defect in secretion rather than production, we extracted lipids from pooled, dissected EBs of *bond*^*e03675*^, *bond*^*res*^, and a control PBac line *97a*^*f06215*^, which has the same genetic background. Analysis by thin-layer chromatography did not reveal a notable signal for CH503 in extract from *bond*^*e03675*^ flies, thus indicating a failure of CH503 synthesis rather than secretion (Fig. 1c). Quantitation by gas chromatography mass spectrometry (GCMS) showed a slight but non-significant decrease in cVA levels in *bond*^*e03675*^ flies when compared with the background control line *97a*^*f06215*^ (Supplementary Fig. 2). Interestingly, both UV-LDI MS and GCMS analysis of *bond*^*e03675*^ extract revealed a prominent signal at *m*/*z* 377.27 ([M+K]^+^; Fig. 1d) that was found only in low quantities in *bond*^*res*^ extract. On the basis of the electron impact spectra and exact mass measurements, the molecule is predicted to contain an acetate and to have the elemental composition C_22_H_42_O_2_. We hypothesize that the compound is used for the synthesis of CH503 and, in the absence of *bond*, remains unincorporated. Taken together, these results indicate that *bond* expression in the EB is necessary for CH503 production but not cVA synthesis.

### Bond contributes to male fertility and male rivalry effects

**Figure 2:**
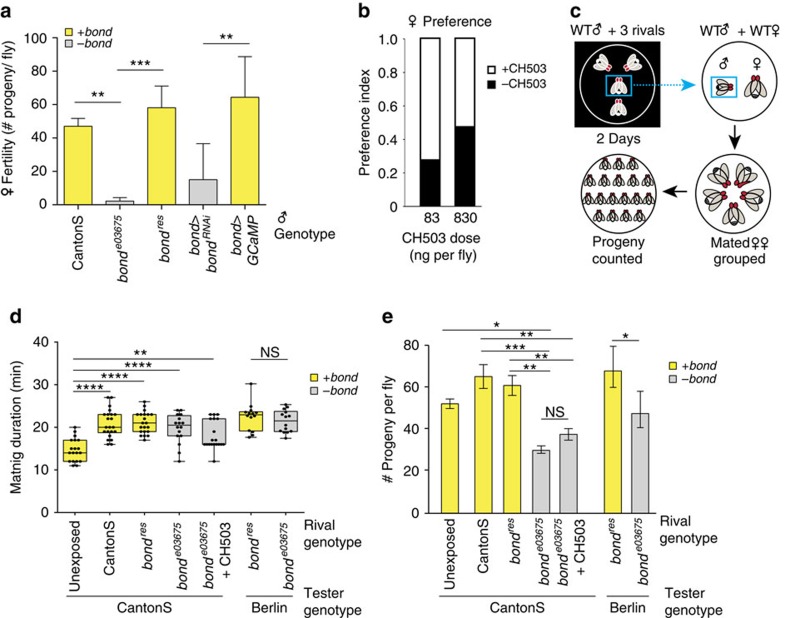
*bond* is essential for male fertility and modulates the ejaculate quality of rival males. (**a**) Females produce significantly less progeny after mating with *bond*-defective males (*bond*^*e03675*^ and *bond>bond*^*RNAi*^) compared with controls (CantonS, *bond*^*res*^ and *bond>GCaMP). bond>GCaMP* is used as a control for transgene load. Bars represent mean±s.d. of three replicates consisting of five females each; ANOVA with Tukey's multiple comparison test, **P<0.01, ***P<0.001. (**b**) Females do not exhibit a significant preference when given a choice to court with *bond*^*e03675*^ males perfumed with hexane or CH503 at a dose of 83 ng (*P*=0.1, *n*=22, Fisher exact probability test) or 830 ng (*P*=0.4, *n*=34) although a medium-sized effect is observed at the lower dose (*r*=0.26). (**c**) Schematic illustrating the design of the rivalry assay. Wild-type (WT) males (blue box) are placed with rivals in the dark for 2 days. The tester is removed and mated with a WT female. Mated females are grouped (five per vial) and progeny counted. (**d**) Male mating duration is not affected by rival genotype. However, the absence of pre-exposure to rivals reduces mating duration. Bottom and top of box plot represent the 25th and 75th percentiles, middle line represents the mean and ends of whiskers represent the minimum and maximum values. ANOVA with Kruskal–Wallis test, *P<0.05, **P<0.01, ***P<0.001, ****P<0.0001, *n*=19–20. (**e**) Males from CantonS and Berlin strains that are exposed to *bond*^*e03675*^ male rivals before mating produce fewer progeny. Bars represent mean±s.d. of three replicates consisting of five females each; ANOVA with Tukey's multiple comparison test, *P<0.05, **P<0.01, ***P<0.001. ANOVA, analysis of variance, NS, not significant.

The product of the *bond* gene was previously described as a VLCFA elongase that is necessary for cytokinesis of *Drosophila* spermatocytes^20^. When tested for fertility defects, both *bond*^*e03675*^ males (1.33±1.24 progeny per fly) and males in which *bond* expression is reduced using RNAi (*bond>bond*^*RNAi*^; 15.03±12.46 progeny per fly) showed low fertility (Fig. 2a). Since *bond* contributes to both spermatogenesis and male pheromone production, we tested the possibility that CH503 may be used as a fertility cue by females. Given a choice between control *bond*^*e03675*^ males and males of the same genotype perfumed with either 83 or 830 ng of CH503, females did not exhibit a statistically significant preference for the perfumed fly. However, there was a medium-sized effect (Pearson's *r*=0.26) resulting from the 83 ng per fly treatment compared with a small-sized effect (Pearson's *r*=0.06) resulting from the 830 ng per fly treatment (Fig. 2b). These results indicate that CH503 at an optimal dose modulates female preference.

Male fertility can also be influenced by social conditions. Previous studies have shown that across numerous taxa, males alter their mating behaviour and sperm allotment in response to rivals^21^,^22^,^23^. In *Drosophila*, a combination of sensory cues underlies the rivalry effect including visual and chemical signals^24^. To test whether *bond* and its biosynthetic products contribute to rivalry-induced modulation of fertility, we exposed males from wild-type strains CantonS or Berlin to three rival males of the same genotype before mating (Fig. 2c). Consistent with previous work^25^,^26^, mating duration increased with prior exposure to rivals, regardless of rival genotype (Fig. 2d). Surprisingly, males of both WT strains exposed to *bond*^*e03675*^ males produced fewer viable offspring when exposed to rivals with functional *bond* (CantonS and *bond*^*res*^ males; Fig. 2e). The decreased fertility was not the result of a shorter mating duration since copulation time was not different between each genotype in the rival-exposure conditions. Fertility increased slightly by exposing CantonS males to *bond*^*e03675*^ males perfumed with synthetic CH503 (effect size, Pearson's *r=0.59*) but was not fully rescued suggesting that other *bond*-related sensory cues are used for assessing rivals.

### Tissue-specific expression of *bond* splice variants

**Figure 3:**
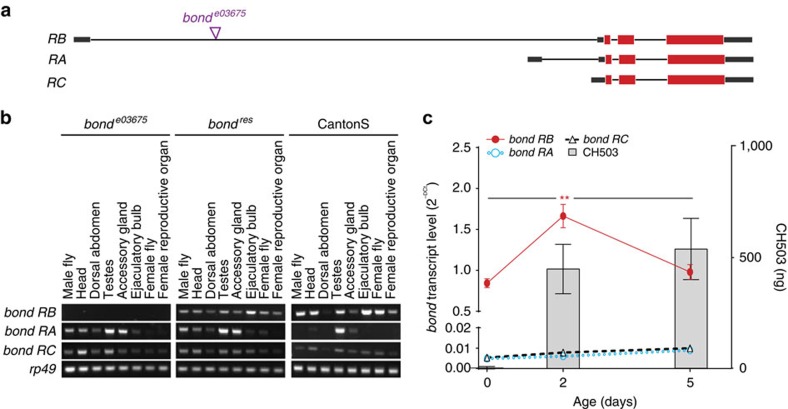
*bond RB* transcript expression in the ejaculatory bulb is age dependent and necessary for CH503 production (**a**) Genetic organization of the *bond* locus and site of PBac-element insertion predicts three transcripts: *RA*, *RB* and *RC.* Purple triangle: site of PBac insertion in *bond*^*e03675*^ flies; grey boxes: non-coding exons; and red boxes: coding exons. (**b**) Semi-quantitative PCR with reverse transcription reveals differential expression of the *bond* transcripts in adult tissues from males and females. Of the three transcripts, *RB* is most highly expressed in the EB of CantonS and *bond*^*res*^ males and was not detected in *bond*^*e03675*^ flies. The *rp49* gene was used as a loading control. (**c**) Quantitative real-time PCR and HPTLC analysis of EB extracts from CantonS males at 0, 2 and 5 days old. Each point represents mean±s.e.m.; ANOVA with Kruskal–Wallis test, **P<0.01, *n*=3. For CH503 quantitation, bars represent mean±s.e.m., three replicate extracts from 20 EBs each. ANOVA, analysis of variance.

Given the diverse functional roles of *bond* in pheromone production and male fertility, we investigated the genetic regulation of *bond* expression in various tissues from adult males and females. The gene is predicted to produce three transcripts, *RB*, *RA* and *RC*. Each transcript encodes an identical protein product but differs in the first non-coding exon (Fig. 3a). *RB* is the primary transcript in the EB of CantonS and *bond*^*res*^ males, with expression levels nearly two orders of magnitude greater than the other transcripts (Fig. 3b,c). In contrast, the *RA* transcript is more highly expressed in other male reproductive organs (testes and accessory glands). In *bond*^*e03675*^ males, the PBac sequence was inserted within the intronic region of *RB* resulting in a loss of *RB* expression in the EB and other tissues (Fig. 3a). Notably, *RA* and *RC* expression could still be detected in the EB and throughout the fly (Fig. 3b). Thus, on the basis of the high relative levels of *RB* expression in the EB and the observation that *RB* expression alone is lost in the EB of *bond*^*e03675*^ males, we conclude that the product of the *bond RB* transcript is essential for CH503 production. In females, the *RB bond* transcript was evident in the reproductive organs, consistent with previous findings^20^. A long non-coding RNA, *CR44062*, is predicted to overlap with the *RB* region but the transcript was not detected in wild-type or transgenic flies and is thus, unlikely to contribute to CH503 production (Supplementary Fig. 3).

### Age-dependent increase in *bond* expression and CH503

To determine if *bond* expression is correlated with sexual maturity, we compared CH503 amounts and *bond* transcript levels from flies at different ages (Fig. 3c). Production of CH503 increases with age: recently-eclosed males produce only trace amounts of the pheromone in the EB while sexually mature males produce approximately 533.7±137.3 ng (Fig. 3c). Analysis by quantitative PCR (qPCR) of EB extracts reveal a spike in *RB* transcript expression at 2 days old, concomitant with a rise in CH503 levels, and a subsequent decrease at 5 days old.

### Bond expression in reproductive organs

**Figure 4:**
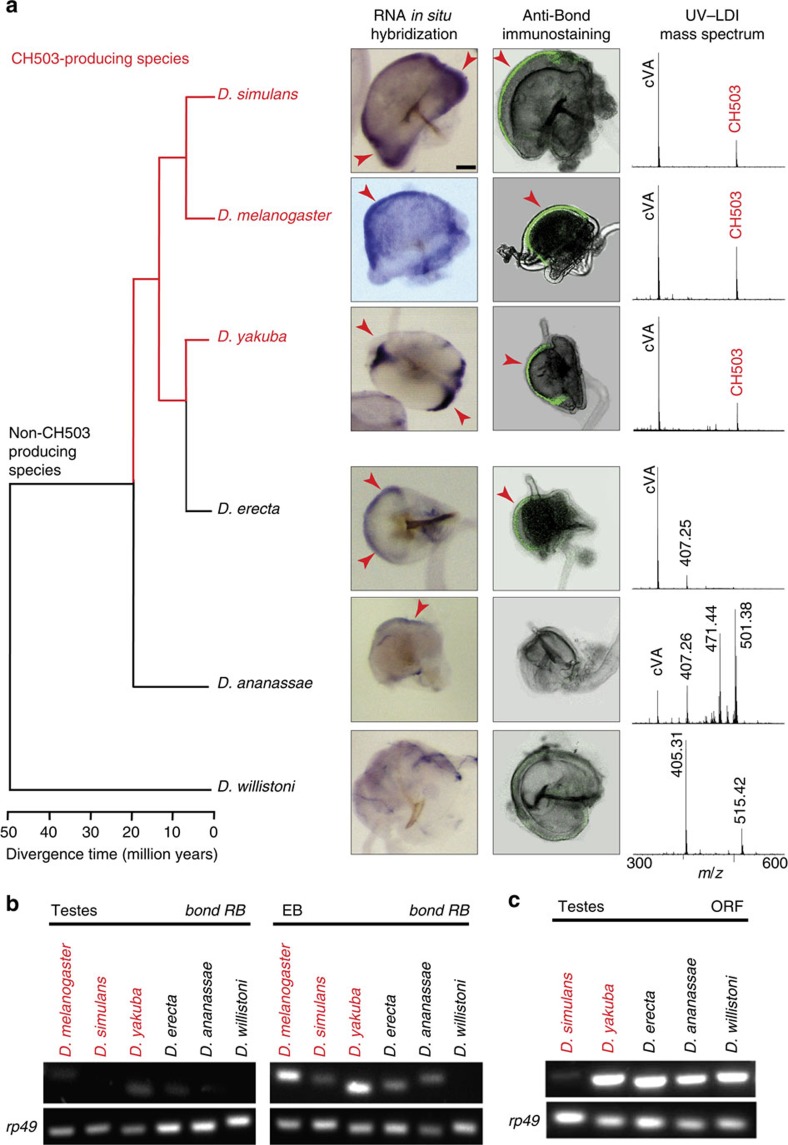
Comparative analysis of *bond* expression in male reproductive organs. (**a**) First column: linear parsimony diagram depicting relations of the drosophilid species used in this study^51^,^52^. CH503-producing species are highlighted in red. Second column: RNA *in situ* hybridization of *bond* transcript in the EB of adult males. Arrowheads indicate positive signal in the epithelium (in purple). Third column: arrowheads indicate positive anti-Bond immunostaining (in green) in the EB. Fourth column: UV-LDI mass spectra from the EB reveal that the production of CH503 is limited to *D. simulans*, *D. melanogaster* and *D. yakuba*. Other unidentified lipids are observed in the EB of *D. ananassae* and *D. willistoni*. Scale bar, 50 μM. (**b**) PCR amplification of the *bond RB* transcript from EB and testes tissue extracts. The *rp49* gene was used as a loading control. (**c**) PCR amplification of the *bond* open-reading frame (ORF) from testes tissue extracts.

Next, we examined the expression pattern of the *bond* transcript and protein in the male testes and EB using RNA *in situ* hybridization (ISH) and immunostaining with an antibody generated to the Bond protein. Both methods revealed expression in the EB wall epithelium (Fig. 4a). Antibody staining also showed labelling of the accessory glands and spermatid heads within the testes, consistent with previous observations^20^ (Supplementary Fig. 4). Positive immunostaining was not observed in the EB of *bond*^*e0367*^ mutants but was evident in *bond*^*res*^ males (Supplementary Fig. 4).

### Bond expression in the EB enables greater chemical diversity

To determine whether the role of Bond in the synthesis of CH503 or other very long-chain fatty acyl pheromones is conserved, we examined expression of the *bond* transcript and protein in the reproductive tissues of different drosophilid species. Previous work has shown that males of *D. simulans* and *D. yakuba* produce CH503 while *D. ananassae*, *D. willistoni* and *D. erecta* do not (Fig. 4a)^12^. As predicted, expression of *bond* messenger RNA and protein is evident in the EB of *D. simulans* and *D. yakuba* (Fig. 4a). Interestingly, the EBs of two non-CH503-producing species, *D. erecta* and *D. ananassae*, also showed positive ISH staining although the signal was much weaker. Positive signal for *bond* by ISH labelling or immunostaining was not observed in *D. willistoni* (Fig. 4b).

We next asked whether *bond* expression in the EB of other species is encoded specifically by the *RB* transcript, as is the case for *D. melanogaster.* Alignment of the *bond* locus identified a putative homologous sequence for the non-coding exon of the *RB* transcript in each of the drosophilids (Supplementary Fig. 5). Semi-qPCR analysis using primers specific to the *RB* transcript revealed expression in the EB of *D. melanogaster, D. simulans*, *D. yakuba, D. erecta* and *D. ananassae* but not *D. willistoni.* Notably, *RB* transcript expression in the testes of all species is comparatively weaker or not detected (Fig. 4b; Supplementary Fig. 4). In contrast, the exon-coding region of *bond* or the protein product is robustly detected in the testes of all species tested, indicating regulation by the *RA* or *RB* transcript splice form (Fig. 4c; Supplementary Fig. 4). The *bond* exon signal from *D. simulans* testes is relatively weak; however, both ISH and immunostaining revealed robust expression of the *bond* transcript and protein (Fig. 4a; Supplementary Fig. 4). Taken together, these results show that *bond* expression is conserved across six drosophilid species in the reproductive organs used for spermatogenesis. However, expression in the EB wall epithelium occurs only in some species and appears to be under control of the *bond RB* splice variant.

## Discussion

The VLCFA elongase Bond was previously shown to be important for spermatogenesis^20^. In this study, we identify a second role for Bond in the production of the sex pheromone CH503. We show also that the products of *bond* are essential for male fertility and modulating the effects of male rivalry on fertility. The gain of a novel transcription initiation site is correlated with the gain of *bond* expression in the EB, thus enabling *Drosophila* males to produce CH503 and other fatty acyl long-chain pheromones.

The Bond elongase belongs to the Elovl family of enzymes, which are conserved from yeast to mammals^20^,^27^ and are involved in the elongation of long-chain fatty acids and VLCFA (longer than 22 carbon atoms)^28^. The VLCFAs are needed for the production of sphingolipids, an integral membrane component of many cell types including spermatocytes, myelin, sebaceous glands and hepatocytes^29^. The substrates for *Drosophila* Bond are not known; however, the *Drosophila* allele is able to rescue *ELO2* and *ELO3* defects in *Saccharomyces cerevisiae*^20^, indicating that Bond is capable of elongating unsaturated long-chain fatty acids to produce fatty acids 20–26 carbons long. The accumulation of an acetylated, monounsaturated C20 lipid in the EBs of *bond*^*e03675*^ flies suggests that Bond could use C20-length fatty acids as a substrate. To produce CH503, one scenario could be that following elongation of C20 by Bond, other biosynthetic enzymes such as a desaturase and fatty acyl reductase add a second double bond and an alcohol, respectively. Heterologous expression of *bond* in bacteria or yeast will be needed to identify the substrates and products.

What is the role of *bond* and its products in male rivalry? Our results indicate that *bond-*associated sensory cues could be used by males to gauge the fitness of their rivals and to adjust the quality of their ejaculate. Sexual competition is a potent modulator of behavioural plasticity across numerous taxa. To maximize reproductive success, male rivalry amongst *Drosophila* causes an increase in males' mating duration and modifies the amount of ovulin, sex peptide and sperm that is transferred to females^24^,^30^,^31^,^32^,^33^. In contrast to previous studies illustrating that male rivalry improves the quality of sperm investment, our results show that social perception can also reduce male ejaculate quality and possibly, quantity. Notably, *bond*^*e03675*^-exposed males have even lower fertility than unexposed males despite a significant increase in mating duration.

The products of *bond* could also designate species identity. In the absence of CH503 and other cues, males may be perceived as a different species or strain. This role for *bond* could explain why females exhibited a slight preference for mating with males perfumed with CH503 compared with mutant males lacking the pheromone. Interestingly, a fecundity suppression effect mediated by social context has been previously reported in *Drosophila* females^34^. Females are infecund when mating with a male of the same strain in the presence of males from a different strain, reminiscent of the Bruce effect observed in rodents^35^. Perhaps, male *Drosophila* are subject to the same phenomenon. It will be important to determine whether the same sensory cues and mechanistic pathways underlie the suppression of fecundity via social perception in males and females. We note that male fertility was fully rescued when placed with *bond*^*re*s^ rivals but only slightly rescued by replacing CH503. Thus, while *bond* and its products are necessary for mediating male rivalry effects and influencing female choice, CH503 is not the only sensory cue needed. Other factors such as the ratio of cVA to CH503, unidentified chemical signals, or a combination of cues from other sensory modalities could signal male reproductive status.

The expression of Bond in the epithelial cell layer of the EB is consistent with its role as a biosynthetic enzyme underlying the production of pheromones. This region of the EB exhibits characteristic ultrastructural features of cells with secretory functions such as a high density of ribosomes and Golgi complexes, electron dense vesicles and a microvilli border along the apical surface^36^. Elo68a, the enzyme underlying cVA elongation, was also localized to epithelial cell layer^16^. While the mass signature for CH503 was detected only in the EB and no other reproductive organs, we cannot exclude the possibility that the accessory glands and/or testes may also produce low levels of CH503 or contribute precursor products. In all CH503-producing species tested, both the *bond* transcript and protein are found in the EB. Unexpectedly, *bond* expression was also observed in non-CH503-producing species *D. erecta* and *D. ananassae*. It may be the case that *bond* contributes to the production of other long-chain fatty acid-based pheromones observed in the EB profiles (for example, the molecule with *m*/*z* 407 found in the EB profile of both species, Fig. 4a).

Several mechanisms have been proposed to underlie the evolution of pheromone biosynthesis enzymes. Most studies support the birth-and-death model of gene duplication, independent gene loss and pseudogenization^37^,^38^,^39^. For example, in drosophilids, the *desat1, desat2* and *desatF* clusters were formed from gene duplication with *desat1* being the most ancient copy^37^,^38^. More recently, *desat2* was independently rendered nonfunctional in the CantonS strain but not the Tai strain of *D. melanogaster*^9^. Studies of desaturases in corn borer moths^40^, indicated a second mechanism involving the fusion of a retrotransposon long interspersed element and desaturase to give new functions. Finally, co-option has been suggested as a third mechanism whereby phenotypic features could diversify. Modification of *trans-* and/or *cis-*regulatory sequences is known to cause expression of a gene in a new tissue and use for a new function^41^,^42^,^43^. On the basis of our observations that *D. willistoni* expresses *bond* in the testes but not EB, it is possible that *bond* first originated as an elongase involved in spermatogenesis. The gain of a novel transcription initiation site (*RB*) allowed expression in another tissue, the EB. Identification and comparative analysis of *cis* elements within the non-coding region of *bond* is needed in order test the hypothesis that *bond* was co-opted from a role in spermatogenesis.

## Methods

### *Drosophila* stocks and husbandry

All flies were raised on standard fly food (autoclaved cornmeal-yeast-sucrose-agar food) at 25 °C with a 12-h-light/dark cycle, unless stated otherwise. The day of eclosion is designated as day 0 for adult fly age. The following lines were used: isogenic CantonS line (#9517), Berlin strain, PiggyBac (PBac) insertion line *bond*^e03675^ (#18181), *97a*^*f06215*^ (#18949), *UAS-GCaMP3* (#32116) and *UAS-mCD8::GFP* (#5137) from Bloomington *Drosophila* Stock centre; *NP4004-GAL4* (#104566; DGRC); and *UAS-bond*^*RNAi*^ (v30179; VDRC). All non-melanogaster species were obtained from the UCSD Drosophila Species Stock Center.

### Transgenic lines

The *bond* PBac insertion rescue line *bond*^*res*^ was generated by crossing *bond*^*e03675*^ males to female flies bearing a ubiquitously expressed transposase (#8283, BDSC). F1 males with mosaic eyes were recovered and backcrossed to *bond*^e03675^. White-eyed flies were collected and backcrossed to *bond*^*e03675*^for at least six generations. DNA sequencing confirmed that *bond*^*res*^ is genetically identical to *bond*^*e03675*^ with only the PBac insertion precisely removed.

To generate *bond-Gal4* lines, a 1.5-kb genomic fragment of the region upstream from the promoter of the *bond RB* transcript was synthesized (Genscript, USA) and inserted into a *pCHs-GAL4* plasmid^44^ using the restriction enzymes KpnI (5′) and SacII (3′). To generate the *UAS-bond* line, the transcript region of *bond* including introns was cloned into a *pUAST-attB* plasmid with BglII (5′) and NotI (3′) (Genscript, USA). The plasmids were injected into *w*^*1118*^ embryos.

### High-performance thin-layer chromatography

EB extract was prepared by extracting dissected bulbs with hexane (Fisher Scientific, New Jersey, USA) followed by brief homogenization with a pipette tip. Chromatographic separation was performed using HPTLC Silica gel 60 glass plates (105631, EMD Chemicals Inc, Darmstadt, Germany), developed with hexane: diethyl ether: acetic acid (66:33:1) and visualized with 0.02% primuline (w/v in 20% acetone; Sigma-Aldrich, St Louis, USA) under long-wave ultraviolet (*λ*=365 nm). To estimate the amount of CH503 from extracts, 0.5–7.0 μg of synthetic CH503 were run in parallel on the same plate. The synthesis of CH503 was previously described^45^. The luminosity of the bands were analysed with Adobe Photoshop (ver. 5.1, Adobe Systems Incorporated, USA) and used to generate a standard curve.

### Ultraviolet–laser desorption ionization mass spectrometry

For intact fly analysis, individual flies were attached to a custom-milled sample plate using double-sided tissue tape (7111, Louis Adhesive Tapes, Thailand). No matrix was used. For Fly-assisted laser desorption/ionization analysis, samples (tissue or extract) were placed onto *Drosophila* wings washed with chloroform: methanol (2:1, v/v) before use to remove endogenous lipid signals. To prevent cross-contamination, each tissue type was placed on a separate wing. More information about the application of Fly-assisted laser desorption/ionization can be found in refs 13, 18, 46. Mass spectra were generated using a QSTAR Elite (AB Sciex, Toronto, CA) mass spectrometer equipped with a modified oMALDI2 ion source (AB Sciex) and a N_2_ laser (*λ*=337 nm) operated at a repetition rate of 40 Hz. Two mbar of N_2_ gas was used create the buffer gas environment for generation of ions. [M+K]^+^ potassium-bearing compounds constitute the major ion species. Spectra were internally calibrated to chitin signals at [M+K]^+^ 242.04, 648.20 and 851.28. Mass accuracy was ∼20 p.p.m. All spectra were acquired in positive ion mode and processed using MS Analyst software (Analyst QS 2.0, AB Sciex).

### Gas chromatography mass spectrometry

Extracts were prepared by placing eight flies in hexane containing 10 μg ml^−1^ hexacosane for 10 min at room temperature. The extract was evaporated with N_2_ in a clean glass vial and stored at −20 °C until analysis. ). Analysis was run in a 5% phenyl-methylpolysiloxane (DB-5, 30-m length, 0.32 i.d., 0.25-μm-film thickness, Agilent) column and GCMS QP2010 system (Shimadzu) with an initial column temperature of 50 °C for 2 min and increment to 300 °C at a rate of 15 °C min^−1^ in splitless mode. Statistical analysis of GCMS results and fertility assays was performed using Prism, v. 6 (GraphPad, La Jolla, CA, USA).

### Quantitative analysis of mRNA transcripts

The dorsal abdomen was dissected by separating the abdomen from the thorax and removing as much of the internal organs as possible without disrupting the oenocytes on the inner dorsal surface. The testes tissues include the testes and seminal vesicles of the male reproductive organ. The accessory gland and EB tissues usually contains part of the ejaculatory duct. The female reproductive organ includes the ovaries and the uterus. Total RNA was prepared using TRIzol Reagent according to the manufacturer's instructions. A concentration between 100–300 ng μl^−1^ was usually obtained. Complementary DNA (cDNA) was prepared using Superscript III Reverse transcriptase (18080-044, Life Technologies, USA). Semi-qPCR was performed with 30 ng cDNA using Taq DNA Polymerase (11146176001, Roche, Switzerland) according to the manufacturer's instructions. The annealing temperature was 58 °C; 25 cycles were performed. See Supplementary Table 1 for primer sequences.

For qPCR, the extracted RNA from the EB was treated with TURBO DNA-free Kit (AM1907, Life Technologies, USA) before reverse transcription reaction (as above). A amount of 5 ng of cDNA was used for qPCR, using KAPA SYBR FAST qPCR Kit (KK4604, KAPA Biosystems, USA) and Applied Biosystems 7900HT Fast Real-Time PCR system. The conditions used were as recommended for in the KAPA SYBR FAST qPCR Kit. Relative transcript levels were calculated as 2^ΔCt^, ΔCt=Ct_act5c_−Ct_sample_^47^. Three biological replicates and three technical replicate of each biological replicate were performed for each time point. Values from each replicate were normalized to levels of the reference gene (*actin5c*) and the mean and s.e.m. calculated from normalized values. An analysis of varinace comparing the normalized levels at each age was performed using Prism, v. 6 (GraphPad, La Jolla, CA, USA). See Supplementary Table 1 for primer sequences.

### RNA *in situ* hybridization

To generate the RNA probe, primers with SP6 (5′-GATTTAGGTGACACTATAG-3′) or T7 (5′-TAATACGACTCACTATAGGG-3′) RNA polymerase promoter sequences tagged to the 5′-end were designed to amplify an approximate 500-bp region of the third exon and used at an annealing temperature of 58 °C. The PCR products were transcribed with either SP6 or T7 RNA polymerase and DIG RNA labelling mix (11277073910, Roche, Germany). Primer sequences can be found in Supplementary Table 1.

The tissues were dissected in phosphate buffered saline (PBS), fixed in PBS:formaldehyde (v:v, 2:1) for 20 min, washed with PBST (0.05% Tween 20 in PBS, pH 7.4) and treated with Proteinase K (50 μg ml^−1^). A amount of 2 mg ml^−1^ glycine was used to stop the Proteinase K reaction, followed by two washes with PBST and refixing for 20 min. Next, the tissues were washed twice in each of PBST, 50% hybridization solution (HS) in PBST and 100% HS (50% formamide, 5 × saline sodum citrate (SSC) buffer, 200 μg ml^−1^ tRNA, 50 μg mL^−1^ heparin, 0.1% Tween-20, pH 6.0). Prehybridisation was performed with 100% HS at 65 °C for 1 h, followed by overnight hybridization with RNA probe (1.3–1.5 μg μl^−1^, denatured for 5 min at 95 °C beforehand). On the next day, the tissues were washed at 65 °C twice in each of 100% HS, 50% HS, 2 × SSC with 0.1% Tween-20 and 0.2 × SSC with 0.1% Tween-20. Tissues were blocked for 30 min at room temperature with 5% normal goat serum (NGS) in PBX (0.1% Triton X-100 in PBS, pH 7.4) before incubating with anti-Digoxigenin-AP (11093274910, Roche, Germany) for 1 h. Following washes in PBX and 0.1% Tween-20 in AP buffer (0.1 M Tris (pH 9.5), 0.2 M NaCl and 50 mM MgCl_2_), the tissues were incubated with NBT-BCIP substrate (B1911, Sigma-Aldrich, St Louis, USA). A brief wash in PBST was used to stop the reaction. The tissues were mounted with 50% glycerol in PBS and imaging was performed with Leica MZ10 F modular stereo microscope.

### Immunostaining

Mouse monoclonal antibodies raised against the peptide sequence LQNGQVGKAY from the Bond protein were generated (Ab-mart, China). Tissues were dissected in PBS, fixed in 4% paraformaldehyde in PBS (v/v) for 30 min at room temperature, washed in PBST and incubated at room temperature for 1 h with the anti-Bond antibody (diluted 1:10,000 in blocking solution consisting of 0.025% sodium azide and 5% NGS in PBST). After washing with PBST, the tissues were incubated with secondary antibody α-mouse 488 (1:1,000, A21202, Life Technologies, USA) for 1 h at room temperature, washed with PBST, and mounted on the slide with VECTASHIELD (H-1000, Vector Lab, CA, USA) for imaging. Imaging was performed with Zeiss LSM5 *EXCITER* Laser Scanning microscope.

### Female choice assay

Six *bond*^*e03675*^ males were placed in a 1.8-ml glass vial (Wheaton, NJ, USA) containing evaporated hexane, 83 ng CH503, or 830 ng (*S,Z,Z*)-CH503 (kind gifts of K. Mori, Toyo-Gosei Company, Japan; synthesis described in refs 45, 48). The flies were vortexed three times with 20-s rest intervals. Following a 1-h recovery period, two males (one of each treatment, differentiated by marking the wings) and one virgin female were placed in a 16 × 9-mm courtship chamber and their behaviour monitored for 30 min. Female choice is defined as the first male with which the female mated. Trials in which females did not mate within 60 min were excluded. Preference index is represented as total trials for one choice (perfumed or non-perfumed) normalized to the total number of trials. Results were analysed using a one-tail Fisher exact test (Vassar Stats: vasssarstats.net), comparing experimental results to null condition (no preference, 50:50 distribution of choices). Effect size was calculated from the odds ratio^49^.

### Fertility assay

Males were isolated from pupal stage in test tubes containing 2 ml of fly food. CantonS virgin females were housed groups of 10. At 5 days old, a single male and virgin female were placed in a test tube for mating. The mated females were kept in food vials in three replicate groups of five flies each. Fresh vials were provided every 2–3 days. The total number of adult progeny from all vials was counted.

### Rivalry assay

CantonS males were isolated from pupal stage as above in test tubes containing 2 ml fly food. At 3 days old, three rival males were introduced. The wings of the rivals were marked for identification. To eliminate visual signals as a rivalry cue, the introduction of rivals and rival incubation was performed in a dark room with red light. After 2 days, the CantonS male was placed in a fresh test tube and allowed to acclimatize to light for an hour. A virgin CantonS female was introduced and the length of copulation scored. Fertility was scored as described for the fertility assay.

For all behavioural assays, *post hoc* power analysis using G*Power 3 (ref. 50) indicated that the sample sizes were adequate to achieve a statistical power of at least 0.8 for an *α*-level of 0.05.

## Additional information

**How to cite this article:** Ng, W. C. *et al.* The fatty acid elongase Bond is essential for *Drosophila* sex pheromone synthesis and male fertility. *Nat. Commun.* 6:8263 doi: 10.1038/ncomms9263 (2015).

## Supplementary Material

Supplementary Information Supplementary Figures 1-5 and Supplementary Table 1
