# Supplementary material for: The fatty acid elongase Bond is essential for Drosophila sex pheromone synthesis and male fertility
Source: Nat Commun. 2015 Sep 15;6:8263. doi: 10.1038/ncomms9263 (PMC4579836; doi:10.1038/ncomms9263)
Supplement: Supplementary Information — Supplementary Figures 1-5 and Supplementary Table 1 [file ncomms9263-s1.pdf]

Supplementary Figure 1.

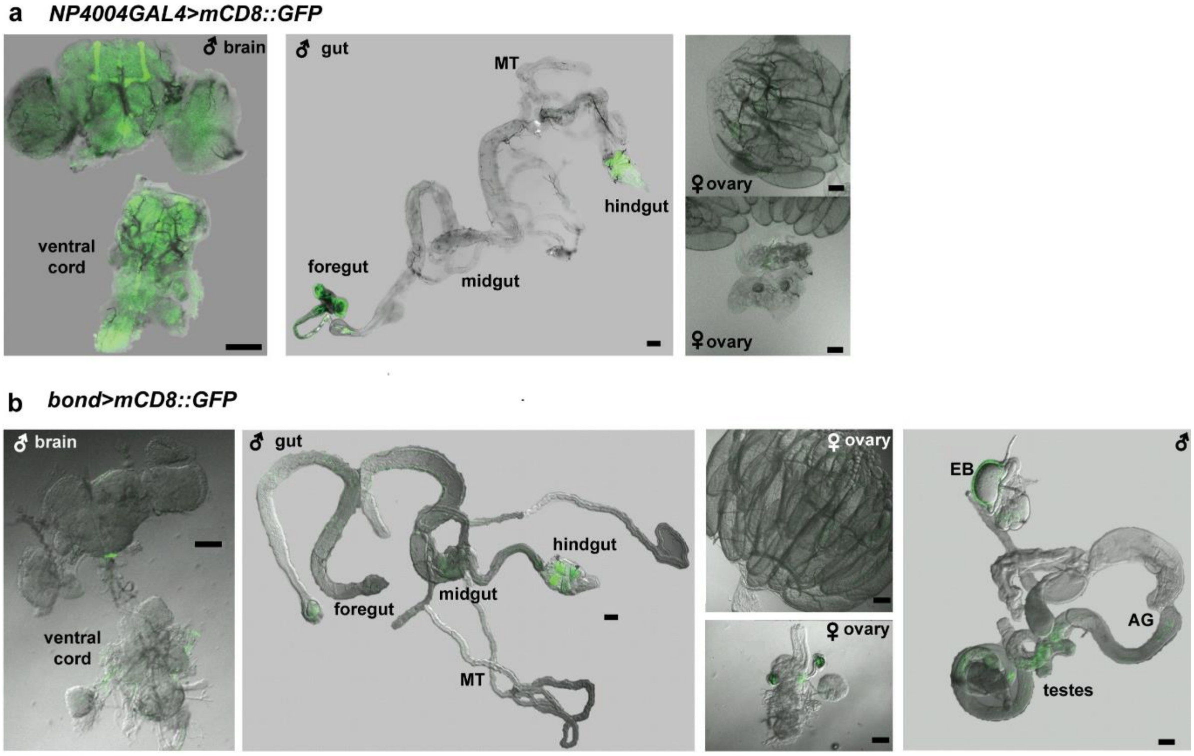

Expression patterns of *NP4004-GAL4* and *bond-GAL4* drivers.

(a) *NP4004-GAL4*-driven green fluorescent protein (GFP) expression is observed throughout the central brain, ventral cord, foregut, and hindgut of males (shown) and females. Diffuse GFP expression is present in the ovaries. Scale bar: 100 µm.

(b) B*ond-GAL4*-driven GFP expression is observed sparsely in the central nervous system and in regions of the foregut, midgut, and hindgut of males (shown) and females. Both female and male reproductive organs show positive GFP signal; EB: ejaculatory bulb; AG: accessory glands. Scale bar: 100 µm.

Supplementary Figure 2.

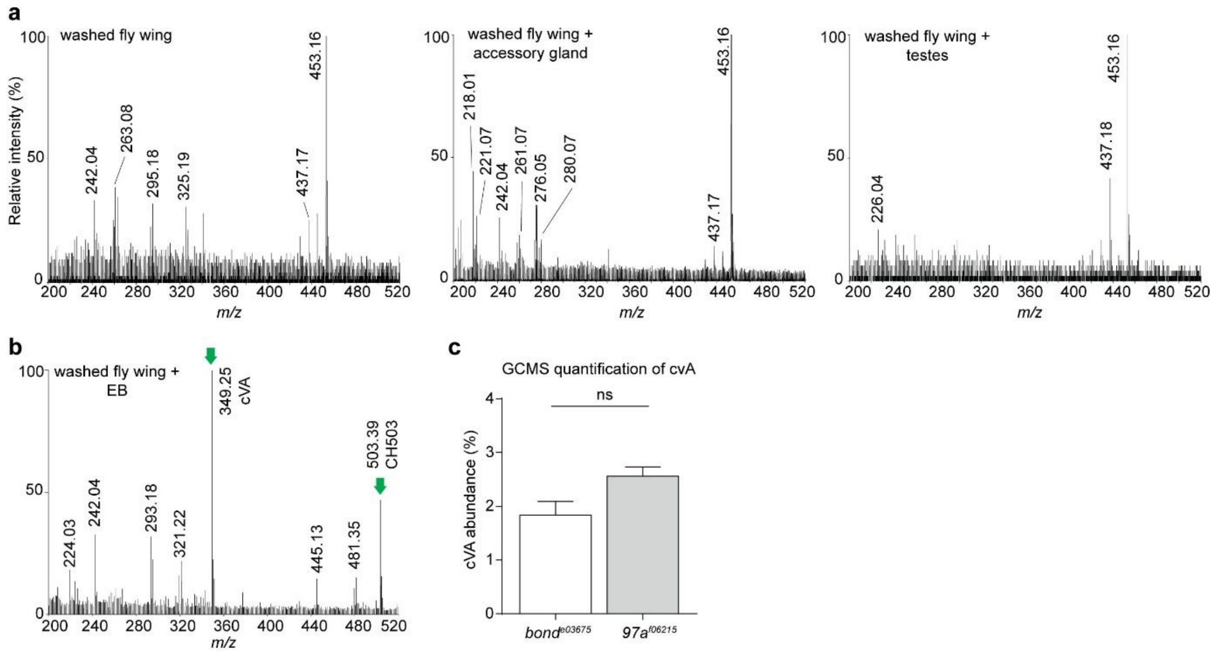

FALDI MS and GCMS analysis of reproductive tissues and whole fly extracts.

(a) Representative FALDI MS spectra from wildtype male accessory glands and testes do not contain signals for cVA (*m/z* 349.24) or CH503 (*m/z* 503.38). Labelled peaks are derived from sugars and chitin ionized from the fly wing.

(b) Representative FALDI MS spectrum from a wildtype male ejaculatory bulb contains signals corresponding to the pheromones cVA and CH503 (green arrows).

(c) GCMS analysis of whole fly extract from $bond^{e03675}$ and $97a^{f06215}$ (control with the same PBac genetic background) shows a slight but non-significant difference in cVA levels; bars represent mean ± SEM, n = 3 replicate extracts from 8 flies each.

Supplementary Figure 3.

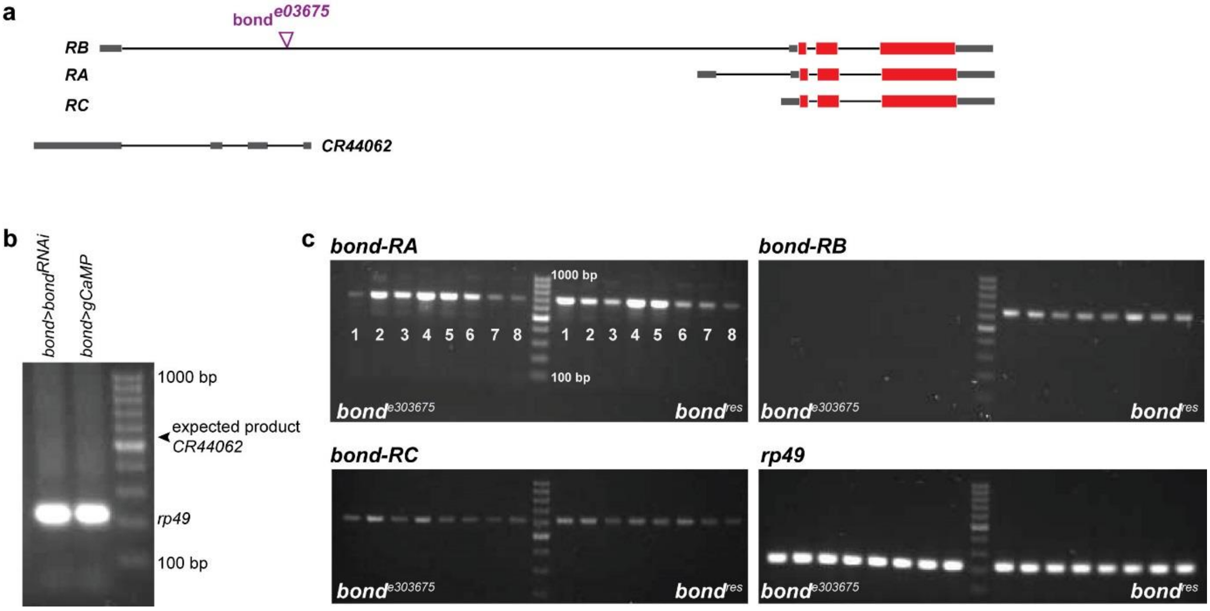

Genetic organization of *bond* and CR44062.

(a) Schematic showing the position of the non-coding RNA CR44062 relative to the *bond* locus. Purple triangle: site of PBac insertion in *bond*^{e03675} flies; grey boxes: non-coding exons; red boxes: coding exons.

(b) Semi-qPCR of *bond>bond*^{RNAi} and control *bond>GCaMP* male flies shows that the long non-coding RNA *CR44062* (expected size of PCR product: 559 bp) is not expressed and hence, is not expected to be affected by suppression of *bond* expression.

(c) Semi-qPCR of *bond* transcripts from adult tissues of *bond*^{e303675} and *bond*^{res} flies. The expected product length of each of the *bond* transcripts is between 612-676 bp. The product length of the *rp49* control is 225 bp. Tissue types in each lane: 1, male fly; 2, head; 3, dorsal abdomen; 4, testes; 5, accessory gland; 6, ejaculatory bulb; 7, female fly; 8, female reproductive organs.

Supplementary Figure 4.

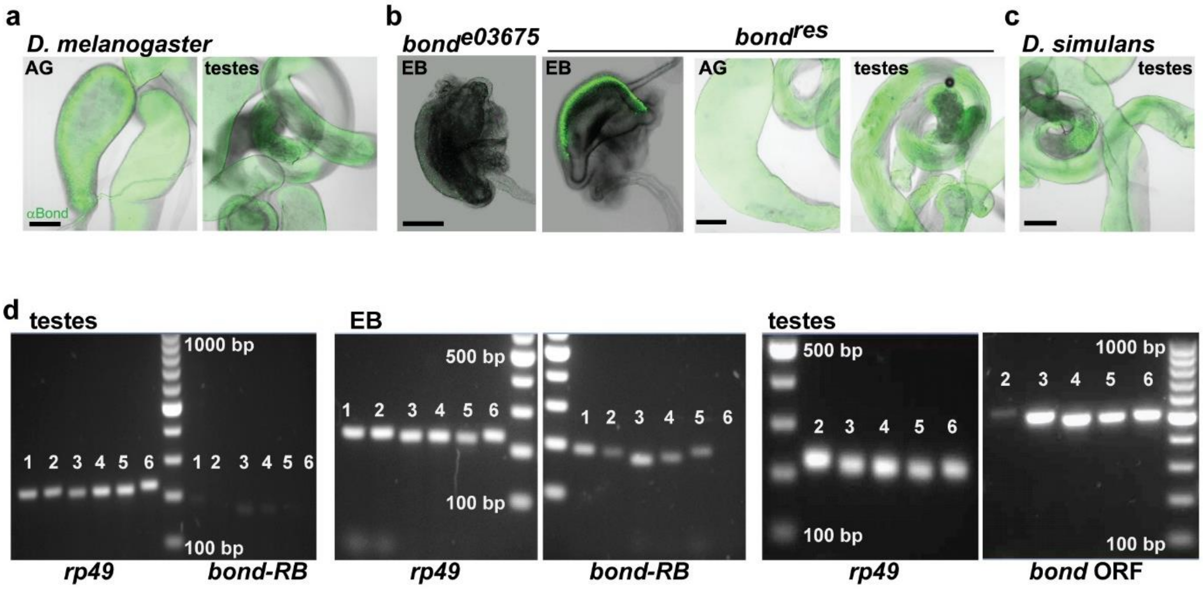

Bond expression in male reproductive organs.

(a) Anti-Bond immunostaining (in green) reveals protein expression in the accessory glands (AG) and testes of *D. melanogaster*. Scale bar: 100 µM.

(**b**) Anti-Bond immunostaining is absent in *bond*^{e03675} EB but restored in the reproductive organs of *bond*^{res} males.

(c) Positive Anti-Bond immunostaining is evident in *D. simulans* testes. Scale bar: 100 µM.

(**d**) PCR amplification of *rp49* and *bond-RB* or *bond* ORF transcripts showing full gels including the DNA ladder. The expected product size for *bond-RB* is between 141-189 bp and between 500-548 bp for the ORF. Species used for each lane: 1, *D. melanogaster*; 2, *D. simulans*; 3, *D. yakuba*; 4, *D. erecta*, 5. *D. ananassae*; 6, *D. willistoni.*

Supplementary Figure 5.

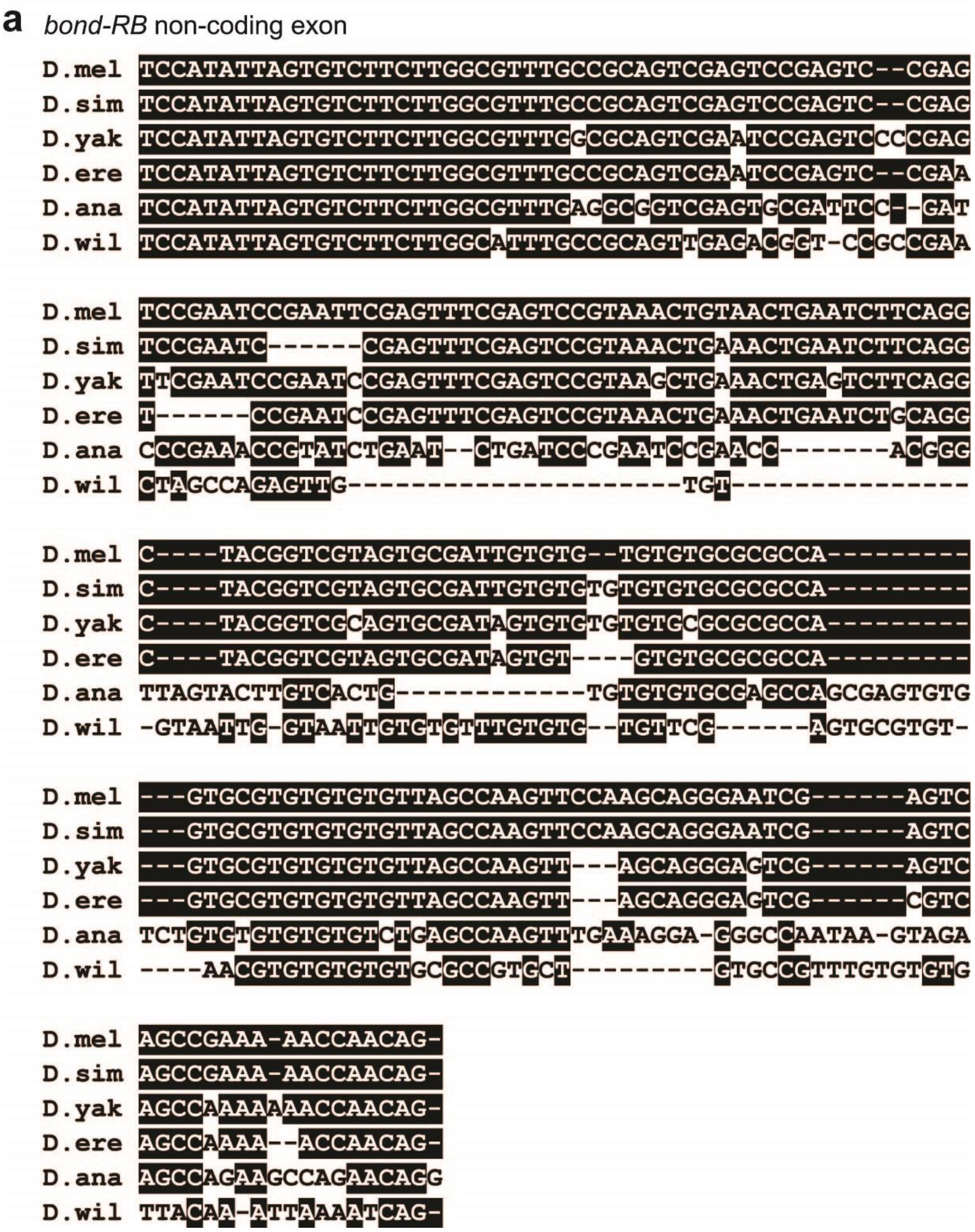

Sequence alignment of putative homologous *bond-RB* regions

(a) ClustalW DNA sequence alignment of the *D. melanogaster bond-RB* non-coding exon to *D. simulans, D. yakuba, D. erecta, D. ananassae,* and *D. willistoni.* Regions highlighted in black are identical to *D. melanogaster*.

Supplementary Table 1. Primer sequences used for semi-qPCR, qPCR, and ISH probe template.

Figure	Target	Forward	Reverse
**3B**	*bond-RB*	5'-CTACGGTCGTAGTGCGATTG	5'-AGGTAGCCCCAGGAGAAGAG
	bond-RA	5'-CCCAACTGGACATCTTCTAGC	5'-AGGTAGCCCCAGGAGAAGAG
	bond-RC	5'-ACGTCGGGCTCTGAATTCTC	5'-AGGTAGCCCCAGGAGAAGAG
	rp49	5'-ATCTCCTTGCGCTTCTTGG	5'-CAAGCCCAAGGGTATCGAC
**3C**	*bond*-RB	5'-AGTGTCTTCTTGGCGTTTGC	5'-GTTGGTTTTTCGGCTGACTC
	bond-RA	5'-TGTGACATTCCCAACTGGAC	5'-CCAACTCCAACCGGAACTC
	bond-RC	5'-CATTCAATATTTCGGCAACG	5'-TTGGCAACTGAAAGAAAGAGG
	actin5c	5'-GAGCGCGGTTACTCTTTCAC	5'-GCCATCTCCTGCTCAAAGTC
**4B**	*Dsim bond-RB*	5'-AGTGTCTTCTTGGCGTTTGC	5'-GTTGGTTTTTCGGCTGACTC
	Dsim rp49	5'-ATCTCCTTGCGCTTCTTGG	5'-CAAGCCCAAGGGTATCGAC
	Dyak bond-RB	5'-AGTGTCTTCTTGGCGTTTGG	5'-TCGACTCCCTGCTAACTTGG
	Dyak rp49	5'-GGTATCGACAACAGAGTGCG	5'-GATCTCCTTGCGCTTCTT
	Dere bond-RB	5'-AGTGTCTTCTTGGCGTTTGC	5'-CTGTTGGTTTTTGGCTGACG
	Dere rp49	5'-GGTATCGACAACAGAGTGCG	5'-GATCTCCTTGCGCTTCTT
	Dana bond-RB	5'-TGTCTTCTTGGCGTTTGAGG	5'-CTGTTCTGGCTTCTGGCTTC
	Dana rp49	5'-GGTATCGACAACAGAGTGCG	5'-GATCTCCTTGCGCTTCTT
	Dwil bond-RB	5'-CATTTGCCGCAGTTGAGAC	5'-GATTTTAATTTGTAACACACACAAACG
	Dwil rp49	5'-TCGTTAAGAAGCGCACCAAG	5'-GAACCTCCAATTCCCGAACG
**S2B**	*CR44062*	5'-GACAGCACGATTTTCACACG	5'-CTTCGACTTGCATCGTATCG
**S4A**	*D.mel*	5'-T7-ATCCTGGGCTACATGCTGAC	5'-SP6-CACGCACTTAGGGTCCTAGC
	D.sim	5'-SP6-GCGCCTGGTTCTACTTCTTC	5'-T7-CTTTCCGGCGTTCATGGTC
	D.yak	5'-SP6-ATGTGATGGCCTCGATCTTC	5'-T7-CGGACTTGGCCTTCTTGTAG
	D.ana	5'-T7-TGATGGCTTCCATTTTCTCC	5'-SP6-GGTCTTGCGGTAGAAGTTGC
	D.ere	5'-T7-TGATGTCGTCGATCTTCTCG	5'-SP6-GGTCTTGCGGTAGAAGTTGC
	D.wil	5'-T7-GGACACGACATTCTTTGTGC	5'-SP6-AATTGGCTGGACTTGGACTG
